# “No judgment, no preaching”: approaches to harm reduction service provision for people who use drugs in North Carolina

**DOI:** 10.1186/s12954-026-01440-y

**Published:** 2026-03-17

**Authors:** David C. Colston, Adams L. Sibley, Elizabeth Joniak-Grant, Hillary L. Mortensen, Monica E. Swilley-Martinez, Brian W. Pence, Vivian F. Go, Shabbar I. Ranapurwala

**Affiliations:** University of North Carolina, Chapel Hill, USA

**Keywords:** Syringe service program, Polysubstance use, Opioids, Communication, Service delivery

## Abstract

**Background:**

Our study examines how harm reduction service providers (providers) throughout North Carolina (NC) provide services to people who use opioids (PWUO), how these service provision strategies align with PWUO preferences, and implications for care.

**Methods:**

We conducted semi-structured in-depth interviews and used a thematic analytic strategy to identify common approaches to service delivery among providers (n = 10), rationales for taking these approaches, and how approaches were received by PWUO (n = 30). To be included in the study, service providers had to provide direct care to PWUO, while PWUO had to be 18 + and be in active opioid use (not as prescribed by a doctor); providers and PWUO had to live in NC.

**Results:**

Providers often allowed PWUO to take the lead in service delivery interactions, asked limited questions about what/how participants use drugs, and rarely offered unsolicited information. Providers believed questioning could feel invasive or stigmatizing to PWUO. They also thought questions were unnecessary, assuming PWUO understood the risks that come with drug use. Providers reported that they would take the lead in interactions to correct PWUO’s misconceptions that could make their use more dangerous, and would, occasionally, offer unsolicited information related to upcoming events or new services, or if PWUO appeared open to receiving more information. PWUO varied in desired approach by providers, based on the established rapport between providers and PWUO, and whether PWUO were in withdrawal during the interaction. Still, PWUO generally felt syringe service programs were a safe space, and many wanted to be asked more about their use so providers could provide tailored information about risks, trends, and safe use.

**Conclusion:**

Providers offer valuable services and safe spaces for PWUO in NC, but should ask program participants’ preferences regarding interaction style to ensure the services provided are aligned with the desires of PWUO to have the maximum possible impact.

**Supplementary Information:**

The online version contains supplementary material available at 10.1186/s12954-026-01440-y.

## Background

Illicit drug-related morbidity and mortality has risen in the United States in recent years, with over 100,000 overdose deaths reported in 2021, 2022, and 2023 [1]. The state of North Carolina (NC) similarly saw massive increases in overdose around this time period, with mortality rates increasing yearly from 22.4 per 100,000 people in 2019 to 41 per 100,000 people in 2023 [2]. This translates to 4,442 overdose deaths, or roughly 12 people a day in NC. The rise in overdose deaths is, in part, attributable to the shifting landscape of substance use in the US. We are firmly in the fourth wave of the overdose epidemic, an era marked by rising fentanyl [3] and intentional and unintentional polysubstance use (i.e., the use of multiple substances concurrently or in close proximity to one another) [4–6]. Individuals engaged in intentional polysubstance use combine certain substances, often an opioid (such as fentanyl) and stimulants (including powder and crack cocaine and methamphetamine) [6, 7].

Unintentional polysubstance use is most commonly caused by taking adulterated drugs [8, 9] with unknown additives. Notable examples include suppliers’ addition of fentanyl or fentanyl precursors to non-opioids (often stimulants) or tranquilizers such as xylazine to fentanyl or other opioids [10–13]. These additives and increasing numbers of counterfeit pills have contributed to a rise in overdose deaths in recent years. Stimulant-involved opioid overdose deaths increased 317% in the U.S. from 2013 to 2019 [14]. This increase continued [1, 15] from 2022 to 2023 despite a 3% decrease in overall overdose deaths in the U.S. during that time. In NC polysubstance use is also common and increasing [16, 17], with three out of every four deaths from 2015 to 2019 having polydrug involvement [16].Thus, understanding and effectively responding to polydrug use is critical to address the current drug overdose epidemic in the United States.

Considering this changing and increasingly dangerous landscape for people who use drugs, harm reduction service providers, such as syringe service programs (SSPs), have evolved to meet the needs of people in active substance use. In addition to the services SSPs offer (e.g., syringes, naloxone) [18–22] there has been an increased focus on drug testing services [23–25] with the distribution of fentanyl and xylazine test strips. [21, 26] This helps PWUO to better understand what they are using in an era marked by concern with synthetic opioids and adulterated supply [8, 27–29]. Still, little is known about how SSPs approach service delivery—more specifically, whether SSP staff use an active or passive approach to communicating risks and protective strategies. Furthermore, given communication strategies can be tailored based on the perceived need of specific groups or individuals [30], it is critical to understand differences between how SSPs communicate about and deliver harm reduction services based on assessed risk profiles for overdose or other drug related harms that may arise due to individual substance use preferences. Preferences may include poly versus mono substance use or the predominant use of certain classes of substances (stimulants, opioids, sedatives)—or changes in the local drug market that may increase susceptibility to unintentional polysubstance use. However SSPs choose to approach service delivery, it is of paramount importance that their strategy does not enact stigma on program participants. PWUO face tremendous judgment in their daily lives, [31] from the general population [32, 33], healthcare workers [34, 35] and in some instances SSP staff [36], which leads to adverse health outcomes [35] and riskier substance use behaviors [36]—the antithesis of what SSPs strive to do.

In this study, we explore harm reduction service providers’ communication strategies and service delivery approaches, including whether providers identify SSP participants’ preferred drug(s) and/or delineate between poly- and mono-substance users, as well as how these factors impact service delivery. We also briefly explore providers’ reasons for their service delivery method, the perceived impact of these approaches, and how this delivery aligns with the wants and needs of people who use drugs.

## Methodology

Qualitative data were collected as part of a mixed-methods study funded by the Centers for Disease Control and Prevention to explore polydrug use in North Carolina. Materials and methods were approved by the University of North Carolina IRB (#23-0808).

### Recruitment

We recruited individuals in active substance use and harm reduction service providers for this study using a purposive sampling approach. We identified relevant harm reduction organizations through the statewide harm reduction networks, and discussions with harm reduction service providers, treatment providers, and individuals in active use. Once identified, we extended invitations to participate. Willing harm reduction organizations also hung flyers and shared information about our study to individuals in active use. Recruitment through NC Syringe Service Programs (SSPs) and word of mouth were the primary ways participants in active substance use were identified and included in the study. Recruitment efforts targeted individuals across North Carolina to capture potential geographic variation in polysubstance use trends and harm reduction service provision.

### Inclusion criteria

Study participants in active use were required to be 18 or older, reside in North Carolina, and have used an illicit/unregulated opioid or prescription opioid not prescribed to them in the past 30 days. Harm reduction professionals had to be 18 years or older and work for an organization that provides drug-related harm reduction services to individuals in active drug use. Exclusion criteria included current imprisonment in a penal institution, or involuntary confinement in a medical facility for psychiatric illness.

### Data collection

In-person qualitative interviews were collected by one team member (DC) from June-November of 2023 with 30 people who use opioids (hereafter people who use opioids, or PWUO) and 11 harm reduction services providers (hereafter providers; 10 of which were retained for analysis, while 1 was removed due to exclusion criteria as they did not provide direct services to PWUO). Written consent was received from all participants and interviews were audio recorded and transcribed verbatim. Basic demographic data were collected on written forms and included questions on harm reduction services offered and substances used in the past 30 days. Separate semi-structured interview guides were used for providers and PWUO, and PWUO completed a visual timeline of key events in their lives. Each participant was offered a $30 gift card. Iterative analyses were conducted during data collection, which allowed for targeted probing of emerging themes in later interviews.

### Data analysis

Transcribed interviews were uploaded to Dedoose v 9.2.007 for analysis. Initial codebooks were developed, and five team members (DC, EJG, AS, HM, MM) index-coded transcripts, grouping like-themes for further analysis [37, 38]. Additional codebooks were then developed and used to broadly capture relevant themes, including intentional and unintentional polydrug use, risks of mono and polydrug use, strategies used to mitigate the potential health impacts of drug use, and experiences with treatment and harm reduction service providers. These codebooks were applied to pre-established index codes, most of which were related to service delivery and the receipt/view/impact of services provided. A portion of all interviews were double coded by two team members (DC, EJG) who then met to establish consensus across codes. DC coded the remaining transcripts individually, using the code definitions established with EJG. All team members reviewed exemplary quotes included in the manuscript, with EJG and AS providing extensive feedback on interpretation and how themes were framed.

We conducted iterative analyses during the interview process of harm reduction professionals and probed more frequently on emerging themes in interviews with providers and PWUO, specifically related to how providers preferred to approach service delivery and why, and how PWUO preferred to receive risk reduction services. Index-coded excerpts from interviews related to service delivery were subsequently reanalyzed and similar themes were synthesized, using Braun and Clarke’s approach to reflexive thematic analysis as a guide [39]. Demographic profiles for participants in active drug use and harm reduction service providers were also summarized and included in Table 1. Pseudonyms were given to all participants, and were paired with corresponding demographic descriptors of sex, classification as provider or PWUO in study sample, and age.

**Table 1:** Demographic profile of participants who use opioids (PWUO) and harm reduction service providers in North Carolina, 2023 (n = 40)

	PWUO (n = 30)		HR service providers (n = 10)	
	N	% (Range)	N	% (Range)
*Gender*				
Female	12	40%	7	70%
Male	18	60%	3	30%
*Race*				
Black or African American	8	27%	–	–
Declined to Answer	1	3%	–	–
Multiple Races	1	3%	–	–
White	20	67%	–	–
Age (Median)	38	(20–71)	38	(23–78)
*Education*				
Less than high school	9	30%	0	0%
High school/GED	9	30%	4	40%
Some college	9	30%	1	10%
College graduate +	3	10%	5	50%
*Employment*				
Unemployed	23	77%	–	–
Part time	3	10%	–	–
Full Time	3	10%	–	–
Declined to answer	1	3%	–	–

## Results

**Fig. 1:**
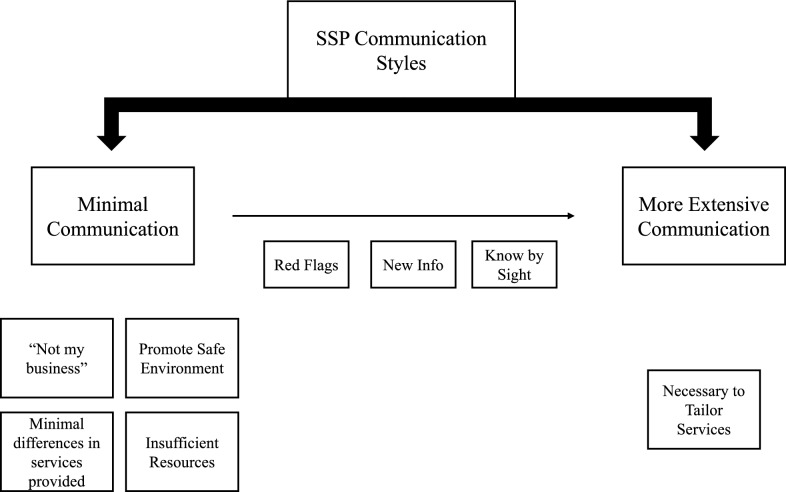
Model for SSP service delivery preferences

Below, we have synthesized in-depth themes regarding providers’ general preference of having PWUO lead service delivery interactions and why this strategy was taken, including: providers’ avoidance of invasive questions when they felt PWUO already knew the risks; minimizing stigma and building rapport to benefit future service delivery; and feeling services had a wide range of applicability, making the need to probe further about what or how people use moot. From there, we explore instances in which providers took a more direct role in leading the interaction, including: when PWUO demonstrated a misunderstanding of a particular substance or harm reduction tool that could make their use more dangerous; if there was a pertinent change that would benefit PWUO to know; or if PWUO seemed open to a more in-depth conversation based on their demeanor or physical appearance. Throughout, service delivery approaches taken by providers are contrasted with the preferences of PWUO. Themes are summarized in the Model for SSP Service Delivery Preferences (Fig. 1).

We interviewed 40 participants: 30 people who use drugs (PWUO) and 10 harm reduction service providers (providers). The majority of PWUO respondents were male (n = 18), and had a median age of 38 years (Table 1). The sample of PWUO was predominantly White (n = 20), followed by Black or African American individuals (n = 8). The majority of PWUO participants had less than a college degree (n = 27) and were unemployed (n = 23). Providers were predominantly female (n = 7), had a median age of 38, and 50% had a college degree or higher.

### Identifying PWUO drug preferences

#### Providers

While some providers try to identify whether SSP participants use poly versus mono-substances and their preferred drugs, many did not. These providers preferred SSP participants take the lead in provider/participant encounters, for any conversations that went beyond basic participant identification (e.g., initials, birthdate) and supply provision (e.g., “What do you need”):

“When you come here, we serve you what you ask for and what you need, and that’s it.” (Susan, Female Provider, 45).

If additional services, information, or help is requested by the SSP participant, providers were ready to lend a hand: “We don’t ask questions unless people…need more help” (Jay, Male Provider, 27).

#### PWUO

The fact that providers frequently do not ask questions outside of those necessary to supply provisions was echoed by several PWUO:

“It's like a Chipotle, man. You go down the line and they're like, you know, "Do you need any of this?" "No." And, "This?" "No." And then you're like, "That, that, that, that." And they ask you how much you need. … Um, and then, you know, everything else is pretty much just watcha need, on the basis, and then we get some food and we're good.” (Tom, Male PWUO, 30)

Tom and Samuel describe the efficient—yet transactional—nature of service provision they have experienced at SSPs, though PWUO often felt they could have more in-depth conversations if they wanted or needed to, “You can actually talk to’em if they have stuff that they can help you with. Like, they’ll try their best to help you” (Matthew, Male PWUO, 36). Some PWUO preferred the strategy detailed by Tom and Samuel—not being asked too many questions or receiving unsolicited information (some of which may already have been available via pamphlets in the SSP common-space), though others were open to answering questions from providers:

“I don’t like sugarcoated shit, you know. Um, tell it like it is. You know, all that’s with me—you know what I’m sayin’? As long as I know I ain’t gonna go to jail for telling you, I’m good.” (Jerry, Male PWUO, 49).

But PWUO recognize preferences may differ between SSP participants—“Me personally, I have no shame” but “that’s just me…I wouldn’t talk about nobody else.” (Damien, Male PWUO, 44). In addition to whether or not the PWUO felt shame, PWUO cited varying levels of rapport with the provider and intensity of withdrawals experienced when accessing the SSP as potential reasons for these differences—

“It just depends, A, on your relationship with the person. I think it's kind of something you build up to. And then other times, people just want their shit and they want to go…And then sometimes people need a friend … I don't know, day to day, wherever they're at in there, you know, if they just got their dope and they need…their stuff to go get high because they're fucking sick, that's all they want to do, you know, is sign something and get the fuck out of there… And then other times, people need to talk.” (Robyn, Female PWUO, 43)

While multiple PWUO clearly state preferences for how providers approach service delivery or communicate with them, several note that there is not one specific approach that would be best for—or at least preferred by—all PWUO using SSP services. Preferred approaches also vary based on the current physical and emotional state an individual may be in—while someone may be open to receiving more information or want more emotional support one day, the same person may need the transaction to be more efficient the next if they are in, say, active withdrawal. This further illuminates the theme that fitting service delivery approaches to meet the needs of specific PWUO should be on a person-by-person and visit-by-visit basis.

### Reasoning/impact of providers not soliciting substance use preferences

#### Avoiding invasive, unnecessary questions—providers

Providers believed participants should lead the interaction for a variety of reasons. For example, providers were concerned asking details about one’s substance use would be too personal or “invasive” (Jay, Male Provider, 27):

“It’s just none of my business. That’s not what we here for. (Susan, Female Provider, 45)

Not asking questions to identify PWUO’s substance use preferences was also a result of some providers’ default perspective that PWUO understand the risks of what they are doing, and additional education on the issue was often unnecessary and paternalistic:

“Most people are aware of—most people know—in those positions know that they're doing risky behavior, and that's [offering information about drug risks] not necessarily going to stop them from doing that. And I don't think—I think maybe if-if they clearly don't understand something or are asking for input, that's different than me just offering like, you know that's not safe, and most people be like, yes, I know that's not safe. Like, you know, I'm doing fentanyl. I know that's not safe. Like, I don't care. That's—I'm trying to be as safe as I can in other ways.” (Jay, Male Provider, 27)

These providers highlight how questioning PWUO about their use might be invasive, and, when taken with the perception that many PWUO are aware that their substance use has potential risks, questioning or providing unsolicited information about their use or risk does not help providers in serving PWUO.

#### (Don’t) talk about my use—PWUO

Several PWUO appreciated that providers did not ask many questions about their substance use because of the invasive, confidential nature of the topic (as highlighted by providers):

“[substance use is] confidential. You know? Uh, they don’t get into that business with nobody. Yeah. That I know of…. I mean, they ask how I’m doing or something like that. You know? But… talking about my use, no.” (Samuel, Male PWUO, 39)

Another PWUO went further, echoing the sentiment that providers do not need to ask about what PWUO are using as there is a mutual understanding of what PWUO are receiving services for and the risks behind it,

“They know what we're using…They're not crazy. They know what they're giving it to us for…They know they're not giving it to us for insulin… you know? They're not crazy.” (Jamie, Female PWUO, 31)

Still, while some PWUO preferred minimal questions or information, others saw value in answering questions that could lead to tailored care:

“Interviewer: Do you appreciate when they don’t ask you about, like, if you’re using certain things? Or would you prefer… that they did ask you, like, what are you using, are you using multiple things….

PWUO: I feel like that's kind of necessary…I kinda feel like it is, yeah. Um, because if you don't know what that person's using, what they're doing, how are you gonna help them?... I think they should ask them.

Interviewer: Okay.

PWUO: Yeah.

Interviewer: And you don't think that would necessarily scare people away?

PWUO: No…. I think maybe some people would be hesitant about it, you know. Um, but I think the majority, I don't think would really mind.” (Tom, Male PWUO, 30)

So, not only did several PWUO feel open to answering more questions or receiving unsolicited information about their use, but a few welcomed it as they felt it might make their use safer.

#### Avoiding questions to build rapport, safe environment

Providers limited questions in an attempt to avoid stigmatizing of PWUO: “No judgment, no preaching, none of that goes on in here” (Susan, Female Provider, 45). This was appreciated by some PWUO given the frequent stigma they felt in their everyday life: “Oh, yeah. I’m judged enough out here by other people. You know?” (Samuel, Male PWUO, 39). Some providers contrasted their approach with the hostile and demeaning questions PWUO have historically faced:

“A big part of harm reduction is, again, like, building trust and respect, and a lot of people are…very used to being told, like, you know that's not safe; you know that you shouldn't do that … so, um, being kind of careful not to talk down to people unintentionally, um, who are spoken down to a lot… about their drug use.” (Jay, Male Provider, 27)

By asking questions that have previously been used in punitive contexts, providers fear they could be rehashing trauma or punishment experienced by PWUO. As Jay points out, providers also try not to elevate themselves, or make themselves appear to be superior to PWUO.

Minimizing questions was also seen as a way to create a safe environment. Creating a safe space is a “priority” (Desmond, Male Provider, 32) of providers because they believe it keeps participants engaged with the SSP long-term.

In contrast, some PWUO expressed that the SSP is already a safe environment, so there is some flexibility in terms of the questions that are asked, as they are coming from a trusted source they know they will not be judged by:

“Yeah, I would take any information that they had. Actually, if there's resources out there that I'm not aware of and they are, I would absolutely, um, be—I'm…approachable anyway. I'm a people person… especially going into the needle exchange. They already know that you're an addict. So you don't have that stigmatism on you. And most of them are addicts, also, you know… or were addicts and whatever. Um, and they're really cool, those people, heh. They're really nice…and they understand. They… totally get it, you know. and that's nice to have somebody that understands instead of judging you…I would definitely be open to any kind of information that was out there…” (Nadia, Female PWUO, 51)

Regardless of preferences for a specific communication or service delivery approach, PWUO and providers uniformly emphasized the importance of compassion, noting its impact on building rapport and service utilization:

“A majority of the time, participants love us. We love them…We just here to make ’em feel good and show ’em a little love…And they show it back.” (Susan, Female Provider, 45)

“PWUO: They make you feel comfortable….You know, I've never been, like, leery about saying anything to 'em. You know what I mean?

Interviewer: Yeah. What do they do that makes you feel particularly, you know, okay with sharing and good with, you know, talking to them and utilizing their services?

PWUO: They're pretty much themselves, you know? They don't try to act like they're better than…anybody…And, you know, they show concern.” (Wayne, Male PWUO, 49)

Moreover, holding space for informal chats where PWUO come in to “just hang out and talk” (Cathy, Female Provider, 38) unrelated to harm reduction are instrumental to developing rapport.

Establishing rapport in a safe environment helps PWUO feel more comfortable sharing information about their lives and drug use, ultimately eliciting the information that might be relevant to harm reduction service provision overall:

“Um, because most people, if they need help, um, they may not ask the first time, but when they come back and they know that you're not going to be judgmental, you're not going to…be nosy. You're not trying to get information about their personal life. A lot of people, I find, are more willing to offer that kind of thing.” (Jay, Male Provider, 27)

“She's not gonna put no pressure on you but if you do want to talk to her, she'll answer any questions you've got. But as far as initiating questions, no. She might initiate some kinda service that she has for you…or she'll kinda, uh, she'll let you know if they're doing any kinda testing or if they, uh, doing any kind of, uh, stuff, like you're doing, uh, interviews…But as far as just bringing up a conversation about the drug use, no. She doesn't do that. But it's the type of relationship you have with her. I feel comfortable with her, will come to her with questions and answers. And then I let her know how I'm doing… You know, I feel free, will talk to her about it with her.” (Larry, Male PWUO, 57)

Finally, some felt that allowing PWUO to lead the interaction builds rapport and provides participants with an outlet if they ever decide to enter a treatment program as that window can be quite narrow—“when people do want help, like, you have a very short window to get them that help” (Becca, Female Provider, 23). Others use established rapport to check up on participants they hadn’t seen in a while:

“We pretty much try to treat everybody, give everybody the same treatment, ask everybody the same questions until they get to know us real good…we've developed good personal relationships with these people. Like, we care about these people. They don't come back; we call and we ask… why didn't I see you? Or, you know, why didn't you let me know you needed something?” (Lindsay, Female Provider, 41)

In contrast to providers who preferred PWUO lead interactions, other providers were more direct:

“We just talk through general conversation. How's it going? You know, um, you know, what are your struggles?...Um, what your barriers to change and whatnot? And, you know, and then that's how all that information comes out. Through a little bit of motivational interviewing…

We do [address the risks of polydrug use]... I do. You know, a lot of outreach workers…who don't have my training as a counselor, they probably don't. But I do. And I always w-warn people of the dangers of using, um, like a benzodiazepine with, you know, fentanyl or, you know, stuff like that.” (Steven, Male Provider, 63)

While motivational interviewing was not frequently discussed by providers in this study—which this participant feels could be due SSP colleagues not having counseling training—this participant uses it as a strategy solicit information about substance use and addressing the risks of certain drug combinations among PWUO. SSP providers who are concerned that taking the lead in interactions would be detrimental to building rapport and relationships with PWUO may also be wary of the sometimes more direct nature of motivational interviewing, though Steven has had success with establishing trust, maintaining long-term relationships with participants, and improving long-term outcomes for PWUO:

“So by me being able to have audience with people at the pre-contemplation stage of change, I'm able to explore, with them, options…um, you know, for when they do wanna change….I'll be like, okay, you don't wanna change now? You good with what you're doin' now, but when you—if there is a time when you want something different, then here's some options. Gimme a call…I would say, I can't tell you the amount of times where I've had people call me three, four, f—six, a year s—uh, later and say, "Hey, you remember me, was talking to me about those changes? I'm ready now." (Steven, Male Provider, 63)

Several providers—that employed different service delivery approaches—identified the importance of this pre-contemplation stage of change, or the ability to capitalize on established relationships to help PWUO enter treatment when they were ready. While these relationships might have been built using different strategies, having a trusted outlet with knowledge about treatment programs and the ability to get them enrolled in a reputable, evidence-based program was essential to their long-term recovery.

#### Lack of resources

One other reason PWUO may need to take the lead in initiating more in-depth conversations about their substance use is that, as the number of PWUO served by SSPs has grown, providers have insufficient capacity to have longer or frequent interactions to all PWUO:

“Everybody—there’s somebody at the front door that makes sure that there are not too many people in the room at the same time…the saddest thing is when you have that many people, you can’t really talk to people.

…when we had 8, 10, 12, 15, 20 people, you know, you could have a conversation. Now we have to work on-on-on deciding if they need a conversation and then …have somebody else come and take-come and take your spot.” (Lavonne, Female Provider, 78)

Even if providers wanted to have conversations with all PWUO, they are resource-constrained and forced to make decisions as to who needs their time. This forces providers to try to identify who would benefit most from their attention, or put the onus of initiating a more extensive conversation on PWUO.

#### Undifferentiated service delivery

Many providers expressed that asking questions to ascertain what a person was using was unnecessary as “95 percent” don’t know “what they getting” (Susan, Female Provider, 45). Asking if PWUO engaged in poly- or mono-substance use was also seen by some as unnecessary as providers assumed PWUO were already in or at risk for polysubstance use, either intentionally or unintentionally:

“Yeah. Yeah. I mean, if they don't tell me, then-then most of the time I'm going to assume that they do [use multiple substances]. Even if it's something as simple as they smoke pot along with whatever…I consider pretty much everyone to be a polysubstance user, unless you specifically say to me, “No.”.”(Lindsay, Female Provider, 41)

Moreover, providers felt the recommendations they gave had wide applicability:

“it's not our job or place to tell people, like, whether or not to use multiple drugs at the same time. Um, that's their decision. What we always tell people no matter what is "Never use alone. Go slow. Start with a small amount, especially if it's a new supply. Um, go slow and be careful. Be aware. Test things. … So those are the things we're gonna counsel everyone to do no matter what. But, yeah, we're not gonna counsel anybody differently” (Desmond, Male Provider, 32)

Providers’ perspective that identifying whether PWUO are in mono- versus poly-substance use as unnecessary due to the broad applicability of services delivered (content provided and approach taken) extends further to include PWUO’s substance of choice:

“I feel like the drug that you're using is not so important. … What fuckin' difference does it make whether you're doing meth or heroin. You're still doing something that's creating all this shit going on in your head and your body. And it's still, you know, like, you're still in danger because of all of it.” (Valerie, Female Provider, 37).

By treating the issues underlying substance use, like trauma, rather than the effects of the specific drug, the providers feel they are better able to target the root of the issue.

### When providers “Lead” the interaction

Despite many providers’ preference to limit questioning and unsolicited education of PWUO, there are times when providers are more likely to probe about drug preferences, knowledge, or provide unsolicited information. These times include when PWUO misunderstand what they are using or are unaware about drug-related risks, changes in the drug supply, or upcoming SSP events. They may also probe based on the demeanor and appearance of the participant.

#### Red flags

One instance in which providers offer unsolicited information to PWUO is if red flags were raised during service delivery:

“There'll be instances where, you know, somebody will come in and say, like—I'll ask them, "Okay, do you-do you need naloxone? Do you need Narcan?”

And they'll say something like, "Nah, I don't do that shit." And I'm like, "Okay." And then there's a conversation of like, "Okay, well, you know that the drug supply is tainted and you know, you need to make sure even as a stimulant user that you have Narcan on you that your friends know—you know. Um, so, we'll have conversations that way.” (Cathy, Female Provider, 38)

While providers often assume PWUO know the risks of the drugs they are using, when it becomes clear that there might be a gap in knowledge, providers view that as a red flag that could ultimately make their use less safe. Some providers who deferred to PWUO during service delivery to avoid feelings of judgement or paternalism even stated they would correct misconceptions to ensure participants know about and can engage in evidence-based harm reduction strategies. In doing so, providers demonstrate their priority of empowering PWUO to make informed decisions related to their use and potentially protect against drug related harms, even though it may make PWUO feel uncomfortable.

#### Breaking news

Providers also proactively offer information to PWUO if there is new, relevant information, related to the local drug supply, services they offer, new harm reduction tools, or to announce upcoming events, like a wound care clinic.

“I mean, they really do a good job at the harm reduction place. You know what I’m saying? I mean, sometimes certain ones don’t really talk to you too much, but if there’s something important, like, uh, watch out for such and such—you know what I’m saying? There’s - there’s whatever drugs going around or something, they’re pretty good. Uh, ’cause he’s reminded me a couple of times, like, “Hey, I know, you probably don’t—probably don’t do this, but—you know what I’m saying? Be careful of it—” “- if you get around it or something ’cause it can kill you or—” You know what I’m saying? Da-da-da, whatever. So, I mean I go there every couple of days. Even if I’m not getting nothing, I just go by there. You know what I’m saying?” (Trenton, Male PWUO, 27)

Proactively providing information about local, recent drug trends was appreciated and helped with retention of this participant, who routinely visited to receive up-to-date information.

#### Know them when we see them

Many providers felt able to identify what service delivery approach is most appropriate for each specific SSP participant,

“I think it’s on a case by case, right? Like, if someone comes in with a hat down, eyes averted, very closed off, we will stick to the bare minimum, no—rare—few questions. “What do you need? We’ll send you on your way.” Um, but those people are becoming less and less. And so, we—I think we just read the room, and we let the participant kinda guide the way. If I’ve—first time, I probably won’t just come right out and—I don't know, maybe I will. I don't know. I think it’s just case by case, really.” (Krista, Female Provider, 36)

The approach providers took in identifying how to provide services for different PWUO was attributed in part, by both providers and PWUO, to the lived experience they have with substance use and dependence:

“Also, like, it takes one to know one. You know what I'm saying? Like, and so a lot of times, you know, I can take—I can pick, uh, somebody out that was using certain drugs about, you know, three miles away. You know, 'cause you just know. You know the signs, you know—you know, you never wanna assume 'cause there also is mental health stuff, you know. Well, it's all mental health, but you know what I mean.” (Valerie, Female Provider, 37)

“I guess when they say you spot it, you got it. [Laughter] You kind of recognize the shit, you know, 'cause you've been there. You know? You can tell by people's behavior. I think—I don't know. Some people I don't even think you have to have a history using drugs. You just got to be good at reading people.” (Robyn, Female PWUO, 43)

By having the perspective of individuals with lived experience, providers are better able to recognize who needs a conversation, which is critical in a resource-constrained environment.

## Discussion

While differences exist in the delivery of harm reduction services to PWUO in North Carolina, many harm reduction service providers prefer PWUO lead the service delivery interaction. More specifically, providers often limit questions, refrain from gauging PWUO knowledge, and do not offer unsolicited information to PWUO. Their reasons are varied. First, providers often think information-sharing is unnecessary as they assume PWUO generally understand the risks associated with substance use. Indeed, PWUO have a general understanding that their substance use carries some risk for overdose [40], though knowledge for risk is imperfect, and sometimes viewed to be more present for other people who use drugs than themselves [41]. Still, there is an understanding between provider and PWUO of a negotiated balance between risk and reward, and the benefit of feeling pleasure, high, or staving off withdrawal may outweigh known risks when it comes to utilizing their drug of choice [42] or known strategies to minimize drug-related injury [43]. Second, providers believe services provided by SSPs have a wide range of applicability regardless of drug(s) of choice or polydrug status. Third, resource limitations mean providers do not have the capacity to talk at length with most who utilize services. Budgetary constraints are common among SSPs [44, 45], and impede service delivery for PWUO [46]. Fourth, providers believed holding back in interactions helped create a safe space for PWUO free of judgment, which they saw as critical to retain and establish rapport with PWUO. They also believed this helped participants feel comfortable discussing their use and linking them to treatment in the future.

Providers generally took on a more direct role in service delivery when certain criteria were met. Providers were more direct if they felt there was a misperception of the local drug supply or harm reduction strategies among PWUO that could lead to more dangerous substance use. They also offered unsolicited information if there were new services offered by the SSP or developments in the local drug supply. Samples from the Opioid Data Lab [47] from June to November 2023 (when qualitative data were collected) show that the median number of substances in each street drug sample (n = 227) was three, and the median for fentanyl-specific samples (n = 114) was five, further demonstrating the scale of drug adulteration in the state. Drug adulteration leaves PWUO exposed to possible unintentional overdose [16, 17] painful wounds [48], intense withdrawals [49], and unwanted highs [50]. SSP providers play a pivotal role in sharing this information with PWUO—what is in the current supply, associated risks, and ways to mitigate adverse effects. Finally, providers were direct if they felt participants were more open to receiving additional information—a trait that both providers and PWUO felt was strengthened by having lived experience prior to or while working at an SSP.

Some PWUO preferred leading interactions with providers, but were okay with fielding additional provider questions meant to improve services or reduce drug-related harms. In these instances, PWUO’s openness to provider-led interactions varied based on the relationship with specific providers and whether the PWUO was experiencing withdrawal at the time of the interaction.

Providers’ attempts to minimize feelings of judgment is reflective of a response to the heavy stigma faced by people who use drugs [31] from a large proportion of the general public [32, 33], individuals in active substance use who may harbor negative sentiment towards themselves [51, 52] or others in perceived “out-groups” based on their drug of choice/method of use/sourcing [53, 54]. Importantly, internalized stigma is a barrier for PWUO to access SSPs services [55], and external stigma from service professionals such as healthcare workers [34, 35] or SSP staff [36] contributes to poorer healthcare outcomes [35] and riskier substance use behaviors [36]. The positive relationships detailed in this study between providers and PWUO and the general comfortability of PWUO when attending SSPs were highly encouraging, and indicate that the prioritization of stigma reduction has had a marked impact on service provision in North Carolina. It is essential to continue building rapport with new and returning PWUO through compassion and informal interaction (beyond the basic provision of supplies).

Moreover, the work that has been done by SSP staff to establish the space as safe may provide an opportunity to better serve PWUO who want more information about their substance use and risk. Specifically, while providers—especially those with lived experience—are capable of identifying appropriate service delivery approaches for PWUO, many deferred to limit questions and/or unsolicited information unless prompted. Given the perception PWUO have of established SSPs as a “safe space”, and the work that has been put in to develop trust between PWUO and provider, providers that are not resource-constrained may benefit from inviting PWUO to weigh-in on whether they would like to have more in-depth conversations about what they are using, risks associated with certain substances or drug combinations, or knowledge regarding risk reduction strategies. This can be done directly by asking about PWUO preferences for the interaction, especially if preferences are unknown to the provider (i.e., there is not an extensive history of interactions between provider and PWUO), ultimately allowing PWUO to opt-into their desired service delivery approach. Or, providers may opt to ‘test the waters’ for service delivery, asking some questions about use, or lightly offering unsolicited information about drug trends/risk reduction strategies and to determine whether to be more or less direct based on the response received. In either case, utilizing the safe space created to collaborate on a service delivery approach would make care even more patient centered [56], and ensure PWUO that want to receive more information are receiving the approach and information most relevant to them.

One limitation of our study is that, because PWUO were recruited primarily through SSPs, findings might not extend to individuals that do not have interactions with harm reduction service providers. Furthermore, there is potential for selection bias as PWUO opted to be in our study, and therefore may be more willing than the general population of PWUO in North Carolina to disclose or be open about their substance use to us or providers. This strategy—recruiting participants through SSPs—was helpful to ensure participants feel safe and relatively comfortable sharing their stories with someone that is at least passively known by the trusted SSP. Disclosing information about substance use is a common challenge and can be met with stigmatizing attitudes [57]. By attending an SSP, PWUO are largely disclosing that they—or someone they know—may use drugs. More research is needed to understand whether differences exist in preferences toward service delivery approaches for people who are more open versus those who are more private about their use. Regardless, participants may have exhibited some social desirability bias given the sensitive nature of the topics being discussed, which also could bias some of our study findings [58]. Finally, we recommend readers are cautious before generalizing findings to all SSPs. SSPs throughout the US vary in philosophy and in what services they can legally provide. In NC, PWUO are not legally obligated to return syringes to receive more [59], in accordance with research that demonstrates the benefits of less restrictive and needs based distribution (as opposed to a 1:1 syringe exchange) [60, 61]. More research is needed on approaches to service delivery—and how they are received by program participants—by region, specific goods provided, and ties to community and faith-based organizations.

## Conclusion

SSPs are seen as trusted, safe environments to receive harm reduction services in North Carolina, and build rapport through compassion and non-judgmental service provision for PWUO. Preferred service delivery approaches vary from person to person. Care should be collaborative and patient-centered to ensure the needs of PWUO are adequately met. More resources should be allocated to SSPs so that, if a more active approach to service delivery is appropriate and preferred by PWUO, they are able to provide those services. Finally, it is critical SSPs to continue to hire and incorporate feedback from individuals with lived experience to guide service delivery.

## Supplementary Information

Additional file1 (DOCX 19 KB)

## Data Availability

Data can be made available upon request from the authors, who will go through proper channels to receive ethics approval and data access.
