# Supplementary material for: “No judgment, no preaching”: approaches to harm reduction service provision for people who use drugs in North Carolina
Source: Harm Reduct J. 2026 Mar 17;23:82. doi: 10.1186/s12954-026-01440-y (PMC13107735; doi:10.1186/s12954-026-01440-y)
Supplement: Supplementary file 1 — Additional file1 (DOCX 19 KB) [file 12954_2026_1440_MOESM1_ESM.docx]

Appendix Table 1. Bank of Additional Notable Quotes

| **Excerpt** | **Section** |
| --- | --- |
| “I don't sit here and-and ask everybody about what drugs they use, 'cause I don't really care.” (Valerie, Female PWUO, 37) | 3.1.A Providers |
| “I just walk in and then I tell ’em. They want your, um, like, some-you know, your initials and, uh, uh, date of birth, and then they ask you what ya’ need, and they check the list, and they put it in a bag for ya’ and send ya’ on your way.” (Samuel, Male PWUO, 39) | 3.1.B PWUO |
| “I mean, we all got different approaches on how we do things. I mean, some people—we are—at-at some point in our world—see, it all depends on what phase you catch a person in...If they're in a phase where they're ge-they're getting sick, they need something, and there you are with a bunch of questions, they're gonna-they're gonna be real nasty. But if they're in a phase where they're not-they're not sick, they're mar—they're comfortable, and they're more apt to answer questions—and the big kick is, what are you offering me” (Christian, Male PWUD, 71) | 3.1.B PWUO |
| “We have the supplies we have. People can get what they need. Um, and so, um, you know, it doesn't really impact us in any way. Like, those folks know what they use, and they know what they need to use safely. Um, and they ask for it, and they get it. So, like, I-I don't think there's any damage being done there, um, in terms of our ability to provide services.” (Desmond, Male Provider, 32) | 3.2.A. Avoiding Invasive, Unnecessary Questions - Providers |
| “PWUD: I think that they ask the questions they do so they know how to help us better.  Interviewer: And do you appreciate that?  PWUD: Yeah.  Interviewer: Okay. That doesn’t scare you off or anything?  PWUD: No.” (Jewel, Female PWUD, 36) | 3.2.B. (Don’t) Talk about my Use – PWUO |
| “Um, I guess someone that-that does ask questions because, you know, they-they'll know how to provide or what to provide for, um, users, you know, because, if they don't know, they can't.” (Wayne, Male PWUO, 49) | 3.2.B. (Don’t) Talk about my Use – PWUO |
| “PWUO: For myself, a lot, I think they should do it more. You know what I’m saying? I think they should do that more often ’cause it really doesn’t—some—I mean some people don’t listen to it, but I think a lot of kids out here that really are clueless, they really do listen to—you know what I’m saying?—what they’re telling. You know what I’m saying? Because it’s true, you know, and they figure it out later on down in life, like, “Damn.” You know what I’m saying? “They were telling me the right thing.” You know what I’m saying?  Interviewer: So you think that wouldn’t dissuade you, or potentially others, from using the services? It might—  PWUO: No, no, no. I think they would use it more. You know what I’m saying? Because it keeps ’em more safe. You know what I’m saying?.”(Trenton, Male PWUO, 27) | 3.2.B. (Don’t) Talk about my Use – PWUO |
| “I mean, the thing is, like, people have been—for decades, people have been asked about their drug use, and it is never a friendly question, you know? Um, so we—you know, I generally just like to avoid sounding at all like all the people that have taken advantage of folks forever, you know? Whether it's a job or it's a legal thing or it's the police, like, answering in the affirmative is always, like, you're getting fucked in some way or the other.” (Desmond, Male Provider, 32) | 3.3.C. Avoiding Questions to Build Rapport, Safe Environment |
| “Interviewer: And do people come and share more over time?  Provider: Oh God, yes. [Laughter] Yes. Yes.” (Cathy, Female Provider, 38) | 3.3.C. Avoiding Questions to Build Rapport, Safe Environment |
